# A natural language processing pipeline for identifying pediatric long COVID symptoms and functional impacts in freeform clinical notes: a RECOVER study

**DOI:** 10.1093/jamiaopen/ooaf089

**Published:** 2025-09-04

**Authors:** H Timothy Bunnell, Cara Reedy, Vitaly Lorman, Ravi Jhaveri, Andrea Rivera-Sepulveda, Katherine S Salamon, Payal B Patel, Keith E Morse, Mattina A Davenport, Lindsay G Cowell, Levon Utidjian, Dimitri A Christakis, Suchitra Rao, Marion R Sills, Abigail Case, Eneida A Mendonca, Bradley W Taylor, Jacqueline Rutter, Aaron Thomas Martinez, Rebecca Letts, L Charles Bailey, Christopher B Forrest, RECOVER Consortium, Iván Diaz, Rachel Kenny, Jasmin Divers, Lorna Thorpe, Hannah Mandel, Jennifer Truong, Shannon Wilneff Wuller, Ravi Jhaveri, Marc Rosenman, Suchitra Rao, Sara J Deakyne Davies, L Charles Bailey, Christopher B Forrest, Nathan M Pajor, Jyothi Priya Alekapatti Nandagopal, Bradley W Taylor, Alexander Stoddard, Kelly J Kelleher, Yungui Huang, H Timothy Bunnell, Marion R Sills, Thuy Le, Dimitri A Christakis, Daksha Ranade, Alan Schroeder, Keith E Morse, Mollie R Cummins, Ramkiran Gouripeddi, Lindsay G Cowell, Phillip Reeder

**Affiliations:** Biomedical Research Informatics Center, Nemours Children’s Health, Wilmington, DE 19803, United States; Biomedical Research Informatics Center, Nemours Children’s Health, Wilmington, DE 19803, United States; Applied Clinical Research Center, Children’s Hospital of Philadelphia, Philadelphia, PA 19104, United States; Division of Infectious Diseases, Ann & Robert H. Lurie Children’s Hospital of Chicago, Chicago, IL 60611, United States; Division of Emergency Medicine and Urgent Care, Nemours Children’s Health, Orlando, FL 32867, United States; Department of Anesthesiology, Perioperative, and Pain Medicine, Stanford University School of Medicine, Menlo Park, CA 94025, United States; Department of Neurology, Seattle Children’s Hospital, Seattle, WA 98105, United States; Department of Pediatrics, Stanford University, Palo Alto, CA 94305, United States; Center for Child Health Equity and Outcomes Research, Abigail Wexner Research Institute at Nationwide Children’s Hospital, Columbus, OH 43205, United States; Department of Health Data Science and Biostatistics, O'Donnell School of Public Health, UT Southwestern Medical Center, Dallas, TX 75390, United States; Applied Clinical Research Center, Children’s Hospital of Philadelphia, Philadelphia, PA 19104, United States; Center for Child Health, Behavior and Development, Seattle Children’s Research Institute, Seattle, WA 98105, United States; Department of Pediatrics, University of Colorado School of Medicine and Children’s Hospital Colorado, Aurora, CO 80045, United States; Department of Research, OCHIN Inc, Portland, OR 97228, United States; Pediatrics and Emergency Medicine, University of Colorado Anschutz Medical Campus, Aurora, CO 80045, United States; Department of Pediatrics and Rehabilitation Medicine, University of Pennsylvania and Children’s Hospital of Philadelphia, Philadelphia, PA 19104, United States; Division of Biomedical Informatics, Cincinnati Children’s Hospital Medical Center, Cincinnati, OH 45229, United States; Clinical and Translational Science Institute, The Medical College of Wisconsin, Milwaukee, WI 53226, United States; RECOVER Patient, Caregiver, or Community Advocate Representative, New York, NY 10012, United States; RECOVER Patient, Caregiver, or Community Advocate Representative, New York, NY 10012, United States; RECOVER Patient, Caregiver, or Community Advocate Representative, New York, NY 10012, United States; Applied Clinical Research Center, Children’s Hospital of Philadelphia, Philadelphia, PA 19104, United States; Applied Clinical Research Center, Children’s Hospital of Philadelphia, Philadelphia, PA 19104, United States

**Keywords:** NLP, pediatrics, PEDSnet, RECOVER

## Abstract

**Objective:**

To develop a natural language processing (NLP) pipeline for unstructured electronic health record (EHR) data to identify symptoms and functional impacts associated with Long COVID in children.

**Materials and Methods:**

We analyzed 48 287 outpatient progress notes from 10 618 pediatric patients from 12 institutions. We evaluated notes obtained 28 to 179 days after a COVID-19 diagnosis or positive test. Two samples were examined: patients with evidence of Long COVID and patients with acute COVID but no evidence of Long COVID based on diagnostic codes. The pipeline identified clinical concepts associated with 21 symptoms and 4 functional impact categories. Subject matter experts (SMEs) screened a sample of 4586 terms from the NLP output to assess pipeline accuracy. Prevalence and concordance of each of the 25 concepts was compared between the 2 patient samples.

**Results:**

A binary assertion measure comparing SME and NLP assertions showed moderate accuracy (N = 4133; F1 = .80) and improved substantially when only high-confidence SME assertions were considered (N = 2043; F1 = .90). Overall, the 25 Long COVID concept categories were markedly more prevalent in the presumptive Long COVID cohort, and differences were noted between concepts identified in notes versus structured data.

**Discussion:**

This preliminary analysis illustrates the additional insight into a syndrome such as Long COVID gained from incorporating notes data, characterizing symptoms and functional impacts.

**Conclusion:**

These data support the importance of incorporating NLP methodology when possible into designing computable phenotypes and to accurately characterize patients with Long COVID.

## Introduction

The COVID-19 pandemic has presented substantial challenges to child health and pediatric healthcare, particularly with the emergence of Post-Acute Sequelae of SARS-CoV-2 infection (Long COVID).^1^^,^^2^ The National Academy of Science, Engineering and Medicine defines Long COVID as “an infection-associated chronic condition (IACC) that occurs after SARS-CoV-2 infection and is present for at least 3 months as a continuous, relapsing and remitting, or progressive disease state that affects one or more organ systems.”^3^ Understanding the full spectrum of Long COVID in children requires comprehensive data, much of which can be obtained from the analysis of electronic health record (EHR) data. However, structured data elements in conventional EHR analyses such as diagnosis, billing, and procedure codes or medication records may be inconsistently used and may not capture the nuanced symptomatic and functional status of pediatric patients. This is particularly likely in conditions, such as Long COVID, which do not have a pathognomonic clinical finding or confirmatory diagnostic test and are characterized by a complex interplay of symptoms, functional impacts, and time.^4^

One obvious source of information to supplement structured EHR data is the corpus of physician notes in EHRs. These contain detailed descriptions of a patient’s clinical history, symptoms, and the impact of those symptoms on physical, cognitive, social, school, and work functioning. Several studies have examined the use of natural language processing (NLP) to identify acute and Long COVID symptoms in freeform text notes. Malden et al used a rules-based NLP algorithm to find symptoms of active COVID infection.^5^ They found that supplementing the structured data with NLP data from notes increased the number of identified symptoms and could identify a COVID infection earlier than using structured data alone. Silverman et al compared 2 NLP pipelines for identifying active COVID based on the CDC COVID symptom list.^6^ The first used a word2vec model trained on medical notes to expand the symptom list, while the other used SPACY to match symptoms with entities in the text. Others have focused on the symptoms of Long COVID. The PASCLex study by Wang et al provided a lexicon of many symptoms and synonyms and used a rule-based approach to search notes for those symptoms.^7^ Liu et al compared a rule-based named entity recognition (NER) tagger and a BERT deep learning NER model on finding Long COVID symptoms in notes from 3 sites and found that the rules-based tagger performed better, though they had a small dataset available for training; they also highlighted the need to train both models on notes from multiple sites.^8^

These studies generally used data from one site or health system; while Liu et al used notes from 3 sites, most came from one.^8^ Because language usage may differ between sites, a model trained on notes from one site may not translate well to other sites. Additionally, they did not focus on a pediatric population, which may have different symptom expression and functional impacts, particularly in young children.^9^

The current study extends this work to a large multi-center collection of notes from pediatric patients using a mixed-method approach that employs both rule-based search strategies and advanced NLP strategies. We present a preliminary comparison of features extracted from medical notes to those extracted from structured EHR elements for 2 patient samples: those meeting a diagnosis code-based computable phenotype for Long COVID and those with confirmed SARS-Cov-2 infection but not Long COVID, and excluding patients with multisystem inflammatory syndrome in children (MIS-C) from both cohorts.^10^ In the following, we describe note selection and preparation, the pipeline implementation, and methods used to evaluate and improve pipeline accuracy.

Our work highlights the inherent challenges in interpreting clinical notes by not only identifying mentions of entities (ie, signs or symptoms) but also accurately assessing their assertion status; for example, whether an entity is mentioned to assert its presence in the patient, or its absence. This approach aims to enhance our understanding of and ability to accurately diagnose Long COVID in pediatric patients by leveraging the full spectrum of information available in EHRs, thus providing a more comprehensive view of patient status and potentially uncovering critical insights into the long-term effects of COVID19 on pediatric populations.

## Materials and methods

### Data collection

All data for this analysis were assembled as part of the National Institutes of Health *Researching COVID to Enhance Recovery* (RECOVER) initiative. The purpose of RECOVER is to improve our understanding of Long COVID, its causes, treatment, and prevention.^11^ For the present analyses, we restricted notes data to those drawn from 12 institutions: Children’s Hospital of Philadelphia, Children’s Hospital Colorado, Ann & Robert H. Lurie Children’s Hospital of Chicago, Cincinnati Children’s Hospital Medical Center, Nationwide Children’s Hospital, Nemours Children’s Health, Seattle Children’s Hospital, Stanford Children’s Health, Oregon Community Health Information Network, University of Utah Medical Center, University of Texas Southwestern Medical Center, and the Medical College of Wisconsin. All institutions participate in PCORnet, the National Patient-Centered Clinical Research Network.

Institute Review Board (IRB) approval was obtained under Biomedical Research Alliance of New York (BRANY) protocol #21-08-508. As a part of the Biomedical Research Alliance of New York (BRANY IRB) process, the protocol has been reviewed in accordance with the institutional guidelines. The Biomedical Research Alliance of New York (BRANY) waived the need for consent and HIPAA authorization.

### Note selection

Notes available for the present analysis spanned the time from January 1, 2020 to September 30, 2022 (see Figures S1 and S2 for the distribution of patients and notes, respectively, over this time frame). Within this timespan, we limited analyses to progress notes written by clinician providers (eg, physicians, nurse practitioners) during outpatient visits by excluding all providers with specialties in an exclusion set (see Supplemental Material: Code sets). We included notes if the note type was missing, assuming they were most likely progress notes. To reduce the potential exposure of identifiable Personal Health Information, institutions deidentified their notes using the TiDE software from Stanford University.^12^

### Study samples

Two cohorts were obtained based on characteristics determined using a previously developed diagnosis-code-based computable phenotype: (1) patients identified as having Long COVID, but not MIS-C; and (2) patients identified as having had SARS-Cov-2 infection with no evidence of diagnosed Long COVID or MIS-C.^10^ Our primary goals were to (a) observe similarities and differences in patterns of feature prevalence for each cohort as derived from notes data and (b) characterize differences in feature prevalence as observed in structured (diagnostic codes) versus unstructured data for both cohorts. By selecting only patients who have had a SARS-CoV-2 infection and not using a negative control group, we anticipated a richer sample of patients with symptoms our pipeline detects.

The computable phenotype identified evidence of Long COVID, either by ICD10CM codes U09.9 or B94.8 or incident diagnoses in any of 23 clusters of Long COVID-associated conditions such as chest pain, headache, fatigue, or respiratory signs and symptoms. Based on this computable phenotype, we selected all patients with notes who met our definition of diagnosed Long COVID, excluding any patients with a diagnosis of MIS-C.

Patients in the acute COVID cohort had evidence for a positive viral or qualifying serologic test (prior to the introduction of vaccines, any serologic test qualified, thereafter, only nucleocapsid tests qualified) for SARS-CoV-2, diagnosis codes for acute COVID, or prescriptions or administrations of Paxlovid or Remdesivir, and excluded those who were members of the Long COVID cohort.^10^

#### Observation window

The observation window for each patient was defined as the 28th to 179th day following their date of earliest evidence of SARS-CoV-2 positivity by lab test or diagnosis (index date). For patients diagnosed with Long COVID who had no prior evidence of COVID-19 in the EHR, we imputed the index date as 59 days prior to their earliest Long COVID diagnosis.

#### Patient matching

To minimize differences between cohorts, we used exact matching on institution and quarter of the index date year with nearest-neighbor propensity score matching on age, race, ethnicity, and sex. Covariate balance following matching was evaluated by calculating absolute standardized mean differences (SMDs) with a threshold of 0.1 for satisfactory balance.

### NLP pipeline

**Figure 1.**
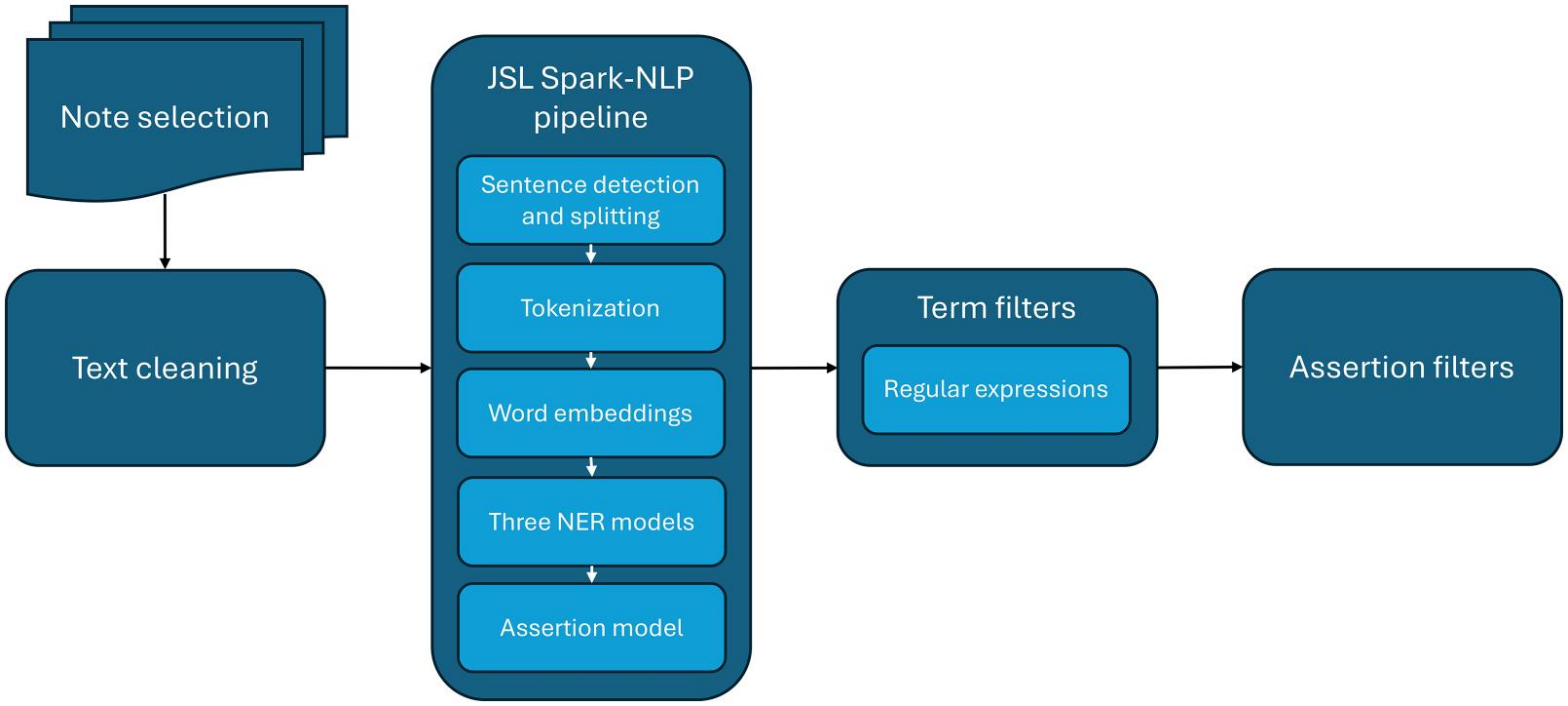
Schematic pipeline structure. Following note selection and cleaning, a Spark NLP pipeline performs NER and assigns assertion status to entities tagged as symptoms. Regular expressions are then used to identify terms associated with 25 Long COVID-related concepts. Those assigned an assertion status of *Present* via a composite assertion model constitute the features assigned to each note.

The pipeline (Figure 1) used openly available as well as commercially licensed Spark-NLP models from John Snow Labs (JSL).^13^ These were supplemented by hand-crafted Regular Expression (RE) rule sets and 1000-element word2vec^14^ embedding vectors trained on 1 million notes randomly selected from the RECOVER pediatric note corpus (code for both the RE and word2vec training and application is available at https://github.com/RECOVER-Coordinating-Center/pediatric_nlp_manuscript_1).

#### Spark-NLP pipeline

Spark NLP is open-source software from JSL, who provide many freely available pre-trained NLP and LLM models. JSL also licenses proprietary healthcare-specific models, some of which were used in this project under a free academic research license. See Supplemental Materials for details of the JSL components used.

#### Term filters

We developed a comprehensive clinical concept typology representing the symptoms and functional impacts that we and others have reported in the literature.^10^^,^^15^ We identified 21 symptom concepts, some of which are generally physically expressed (eg, respiratory symptoms, pain, fatigue) or emotionally expressed (eg, anxiety, depressive symptoms). An additional 4 clinical concepts addressed impairments in physical, cognitive, sleep, and school functioning. We then created a set of expressions—a pediatric Long COVID lexicon—to represent each concept. For example, expressions for *cognitive impairment* included terms such as cognition, brain fog, memory, remembering, forgetting, confusion, attention, and concentration as single terms or in the context of terms like poorer, difficulty, problem, decreased. The process of building the lexicon involved: (1) review of the adult Long COVID lexicon developed by Wang et al^4^; (2) review of the PROMIS patient-reported item banks for concepts with a PROMIS measure; and (3) review of 100 physician notes to ensure that the lexicon adequately addressed the breadth of concept expressions. We further supplemented the lexicon by adding terms identified by concept embeddings formed as an average of the word embeddings for concept-related terms. All terms in the lexicon were formalized as a set of Python regular expressions (REs) identified as “Term Filters” in Figure 1 (see Supplemental Materials for the design of word2vec clinical concept embeddings and concept-specific regular expression term filters).

To facilitate comparison of note-based clinical concepts with structured data, we assembled ICD-10-CM diagnostic code sets for each of the 25 clinical concepts and used those to identify concepts in the structured data.

#### Composite assertion filter

The last stage of the pipeline filtered all the term mentions extracted by the Term filters to remove instances that were not asserted to be present for the patient. Based on the results of our initial pipeline evaluation, we trained a composite assertion model that used ranked votes from the JSL model, a RE assertion model and a word2vec-based assertion model to improve on the accuracy of the JSL model alone in identifying concept mentions as either “*Present*” or “*Absent*.”

#### Concept persistence

To reduce the chance of misattributing Long COVID clinical concepts to patients, we required concepts to be identified in at least 2 notes related to a patient at least 29 days apart to attribute the concept to that patient.

### Pipeline development and evaluation

Pipeline development and evaluation were phased. An initial development dataset provided pipeline output for an extensive evaluation by Subject Matter Experts (SMEs) who were pediatric clinician-scientists, providing “ground truth” data to develop an improved assertion measure. The full study dataset was processed twice by the pipeline, once before, and once after tuning a pre-trained bidirectional LSTM NER component of the pipeline. An additional small evaluation of the final pipeline output was conducted by 2 of the study authors to verify pipeline accuracy. Evaluation data collection leveraged a custom R-Shiny user interface (see Supplemental Materials: ScreenTool). Interrater agreement for SMEs was assessed by Krippendorff’s alpha (α).^16^

### Statistical and data analysis

Demographic variables are presented in terms of means and standard deviations. Comparisons of feature prevalence among and between both cohorts are presented in terms of patient counts and odds ratios adjusted to account for individual differences in the number of encounters and/or progress notes per patient.

Data analyses address 2 primary hypotheses. First, in analyses of notes data, the 25 Long COVID clinical concepts should be more prevalent among Long COVID patients than patients without evidence of Long COVID. Second, we expect to find significant differences between note-derived and structured EHR-derived patient counts in many—but probably not all—of the features. Thus, we directly compared the odds of detecting features in patients using structured versus unstructured data, compared patterns of within-patient concept presence due to data source, and examined the degree of overlap between concepts identified in the 2 data sources. Additionally, we examine site-specific differences in the distribution of the 25 Long COVID clinical concepts. Large differences, if found, may limit the generality of the NLP pipeline.

## Results

### Patients

Of 476 231 pediatric COVID patients in the RECOVER database, notes were available for 204 883, but fewer than 4% (7771 patients) met our current definition of Long COVID. The impact of additional factors such as the availability of correct note type, presence of notes in the observation window, and 1:1 matching process resulted in a matched sample of 10 618 patients. Demographic characteristics of these matched samples are presented in Table 1. These patients contributed a total of 48 287 notes, from which 468 828 concept-related terms were identified.

**Table 1.** Demographic factors used for propensity score matching between cohorts.

	Cohort		
	COVID	Long COVID	SMD
**N**	5309	5309	
**Age at index date, years** (mean (SD))	10.2 (6.7)	10.2 (6.3)	<0.01
**Sex** = MALE (%)	2531 (47.7)	2467 (46.5)	0.02
**Race** (%)			0.08
American Indian or Alaska Native	13 (0.2)	25 (0.5)	
Asian	165 (3.1)	170 (3.2)	
Black or African American	714 (13.4)	800 (15.1)	
Multiple race	213 (4.0)	199 (3.7)	
Native Hawaiian/Pacific Islander	<11 (0.1)	15 (0.3)	
No information	129 (2.4)	131 (2.5)	
Other	474 (8.9)	489 (9.2)	
Refuse to answer	98 (1.8)	95 (1.8)	
Unknown	340 (6.4)	328 (6.2)	
White	3157 (59.5)	3057 (57.6)	
**Ethnicity** (%)			0.0
Hispanic or Latino	1362 (25.7)	1414 (26.6)	
No information	16 (0.3)	12 (0.2)	
Not Hispanic or Latino	3763 (70.9)	3726 (70.2)	
Other	39 (0.7)	39 (0.7)	
Refuse to answer	<11 (0.0)	<11 (0.0)	
Unknown	128 (2.4)	117 (2.2)	

Two other factors, site and quarter year, were matched exactly between cohorts. Standardized mean differences (SMDs) were less than 0.1 for all factors.

We drew an additional “SME Evaluation sample” of 625 patients and notes for pipeline evaluation and tuning prior to running the matched sample study. This random sample included 250 Long COVID patients, 250 COVID patients with no evidence of Long COVID, and 125 patients with no evidence of past/present COVID infection.

### Pipeline evaluation and tuning

Eight SMEs reviewed a total of 4586 entities extracted by our initial pipeline from an evaluation sample of notes from 250 Long COVID patients as well as 139 other (COVID or non-COVID) patients. SMEs found 453 of the entities to be unrelated to the intended concept or the context insufficient to provide an assertion and were dropped from further analyses.

#### Interrater agreement

Considering only feature-relevant tokens assigned an assertion of either *Present* or *Absent*, SME responses were in high agreement (N = 3218, α  =  0.96). Agreement was substantially weaker when considering all 5 assertions (α  =  0.623).

#### Pipeline accuracy and tuning

SME responses were used, first to assess the initial pipeline, and second, to improve overall pipeline accuracy. One of the 3 JSL NER models (the only one that was tunable) received additional tuning following an initial analysis of the study dataset.

##### Assertion model

Two methods were used to reduce the 7 JSL assertions and 5 SME assertions to the binary *Present* or *Absent* assertion: (a) equate all assertions except *Present* with *Absent*; or (b) discard all assertions except *Present* and *Absent*. Results of these are shown in Table 2, which indicates that the latter had much higher accuracy, but resulted in discarding 50% (2090) of the entities, 40% of which (829) were tagged as *Present* by SMEs.

**Table 2.** Confusion matrices showing results of 2 strategies for assigning a binary *Present/Absent* assertion to pipeline output.

SME Assertions	Model assertions			SME Assertions	Model assertions		
	Present	Absent	Row Total		Present	Absent	Row Total
**Present**	904	930	1834	**Present**	904	101	1005
**Absent**	479	1820	2299	**Absent**	168	870	1038
**Total**	1383	2750	4133	**Total**	1072	971	2043
FPR: 0.21; Precision: 0.65; Recall: 0.49;				FPR: 0.16; Precision: 0.84; Recall: 0.90;			
F1: 0.56; Percent correct: 65.91				F1: 0.87; Percent correct: 86.83			

Left matrix assigns all assertions except *Present* to *Absent*. Right matrix discards all assertions except *Present* and *Absent*.

To avoid discarding numerous *Present* assertions, we developed and tuned a composite assertion measure that used RE and wave2vec *Present/Absent* assertion models in combination with all JSL assertions including the many cases that JSL did not provide an assertion because the JSL NER models did not identify an entity. Details of the design and tuning process are described in the Supplemental Materials, and code is provided in our GitHub repository. The results of the final composite measure applied to 2769 entities to which SMEs assigned a *Present* or *Absent* assertion are shown in Table 3.

**Table 3.** Confusion matrix and statistics comparing the output of the pipeline with composite assertion model after being tuned on data from SMEs.

SME assertions	Model assertions		
	Present	Absent	Row total
**Present**	1235	314	1549
**Absent**	202	1018	1220
**Total**	1437	1332	2769
FPR: 0.17; Precision: 0.859; Recall: 0.797; F1: 0.827; *P* < .001			
Overall percentage agreement: 81.4			

##### Entity recognition

An initial pass of the full study note corpus through the pipeline with composite assertion model, revealed that the JSL Clinical NER model failed to detect nearly 17 000 entities that were identified by RE search and classified as *Present* by the composite assertion model. Because entities recognized by the Clinical NER model feed the JSL assertion model, improving the Clinical NER model should impact the composite assertion model as well. Consequently, we fine-tuned the JSL Clinical NER model, leading to a substantial improvement in the output of the NER model compared to a held out set of 1200 examples (see Supplemental Materials for details). Applied to the full input dataset, this led to approximately a 5% improvement in the number of entities captured by the Clinical NER model and a small improvement in the pipeline accuracy.

##### Final evaluation

Four hundred tagged entities were randomly selected from the final pipeline output for evaluation by 2 of the authors. Table 4 provides confusion matrices based on (a) the original untuned pipeline using the JSL assertion model and (b) the output of the final tuned pipeline using the composite assertion model and tuned JSL NER model. Even with this relatively small number of observations (381 of the 400 entities reviewed received an assertion in the authors’ review), the improvement in the tuned pipeline is significant. Moreover, comparison of the 2 matrices suggests that most of the improvement in accuracy was due to correctly assigning *present* assertions to those classified as *absent* by the original JSL model at the expense of adding a single false-positive assertion by the composite model.

**Table 4.** Small sample comparison of the original JSL assertion model (left matrix) with the output of the final composite assertion model (right matrix).

SME		JSL model		SME		Composite model	
		Present	Absent			Present	Absent
Present		127	58	Present		159	26
Absent		62	134	Absent		63	133
Accuracy	Precision	Recall	F1	Accuracy	Precision	Recall	F1
68.5	0.672	0.686	0.679	76.64	0.716	0.859	0.781

The composite model is significantly more accurate than the original JSL assertion model (McNemar’s X^2^ = 6.9767, df = 1, *P* < .01).

### Note-based feature prevalence by cohort

**Figure 2.**
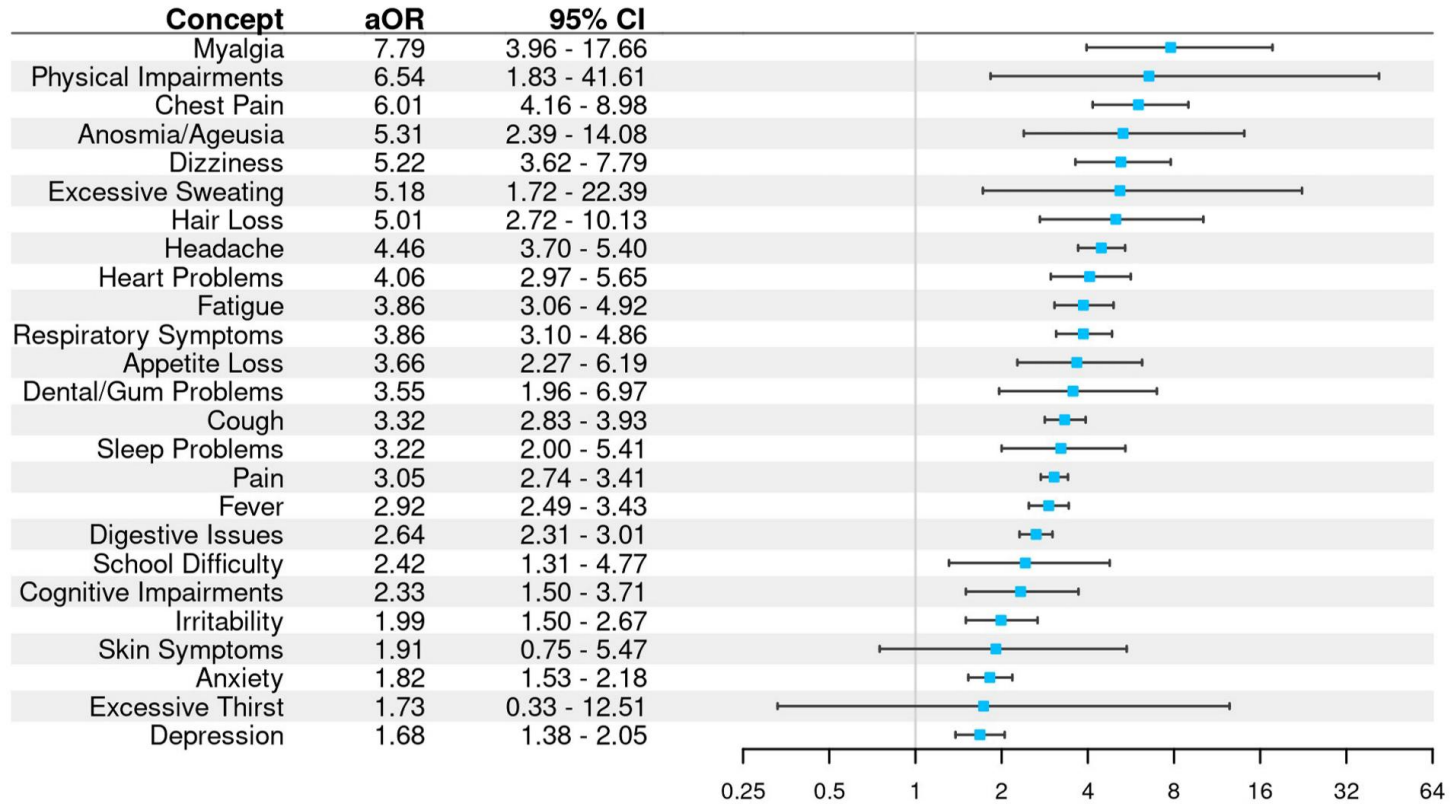
Adjusted odds ratios and 95% CI for each Long COVID feature comparing feature prevalence among Long COVID versus COVID patients. Ratios greater than 1 with CIs that do not extend below 1 indicate the feature was significantly more prevalent in Long COVID patients.

As shown in Figure 2, all clinical concepts were more prevalent in notes data for Long COVID patients, though 2 (excessive thirst and skin symptoms) were infrequently mentioned.

### Structured versus unstructured feature prevalence

**Figure 3.**
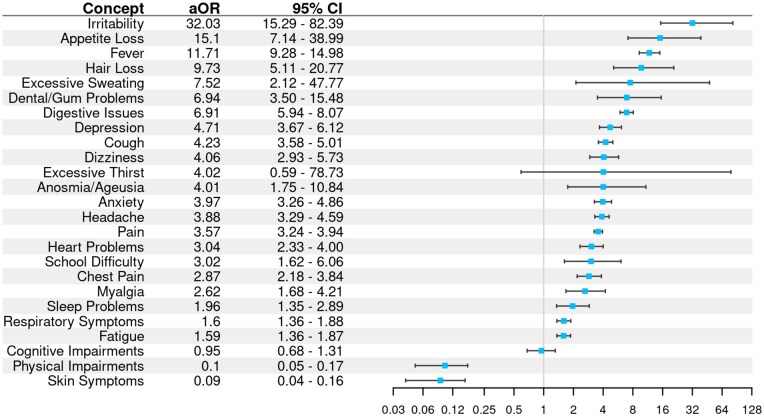
Within-patient adjusted odds ratios and 95% CI for each Long COVID feature comparing prevalence of concepts present in notes versus those present in structured data. Ratios greater than 1 with CIs that do not extend below 1 indicate the feature was significantly more prevalent in notes data. Ratios less than 1 with CIs that do not extend above 1 indicate features significantly more common in structured data.

For all 10 618 patients, we examined the odds of assigning each of the 25 features based on structured EHR codes or NLP mentions asserted to be present (Figure 3). Our structured code sets (one code set for each of the 25 symptom/function categories) were drawn from diagnostic codes. In most cases, concepts were more likely to be observed in notes data than in structured data. However, there were 2 cases (Physical Impairments and Skin Symptoms) in which more patients were identified from structured codes, and the difference for Cognitive Impairment was not significant.

### Per-patient agreement—structured versus unstructured data

**Figure 4.**
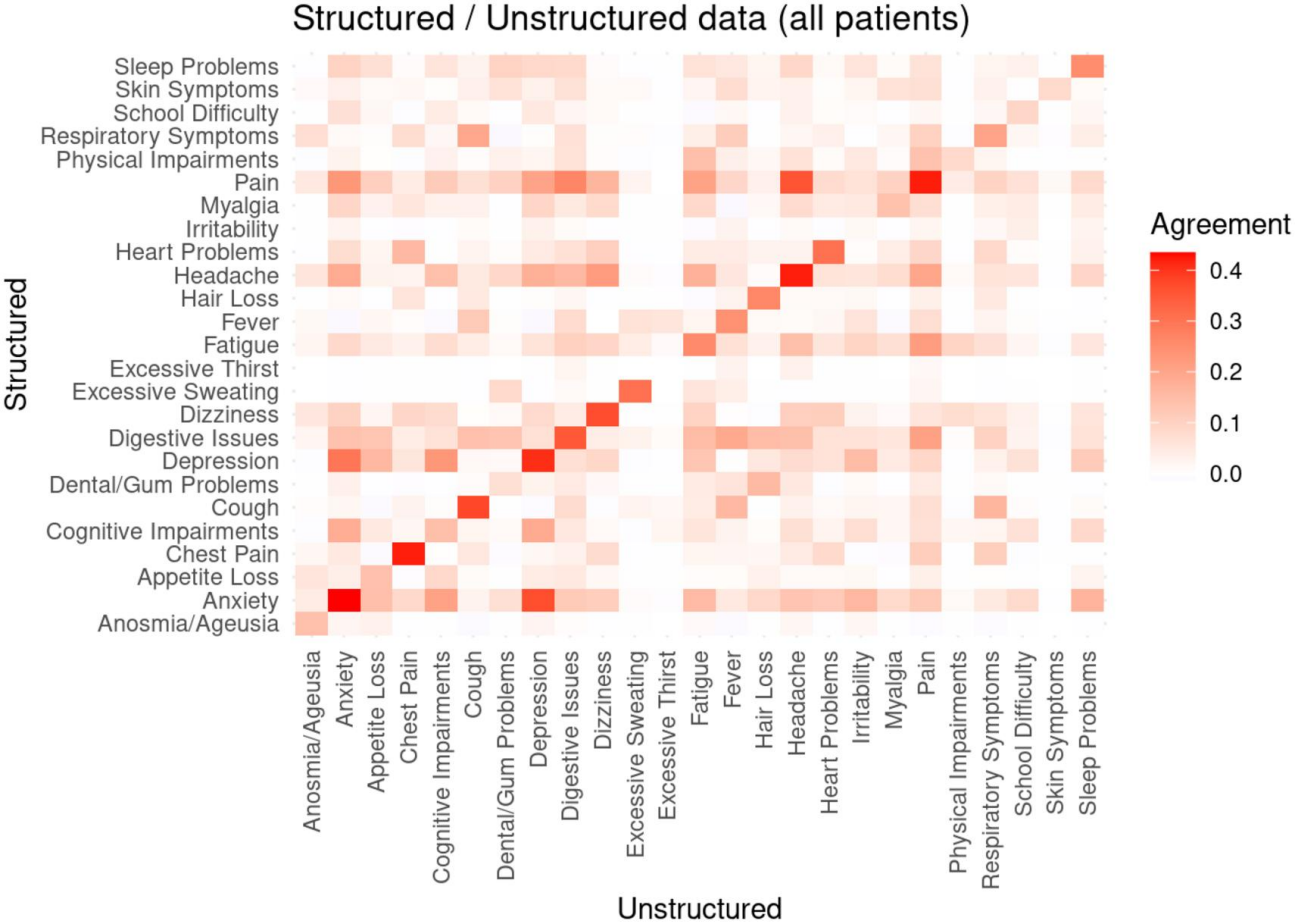
Comparison of feature distribution over patients for features identified through structured versus unstructured analyses.

A crucial question is the extent to which patients identified from structured data are a subset of those identified in the notes data or a disjoint set. We examined this in 2 ways. First, as shown in the Figure 4 heatmap, we calculated the correlation (over patients) between each feature obtained from notes data and each feature identified from structured data. The diagonal in Figure 4 represents the agreement within patients for feature assignment by both data sources, showing at best moderate associations (r ∼= 0.4) for features such as Anxiety, Cough, Depression, and several pain-related features. Several other features, notably Irritability and Excessive Thirst, had near zero correlation.

Off-diagonal cells in the heatmap indicate the correlation between different features from both data sources. For example, Anxiety and Depression appear to be associated both at the (row, column) intersection of (Depression, Anxiety) and (Anxiety, Depression). Note that the relationship is not symmetric: features identified from structured data appear more closely related to those identified from unstructured data than vice versa. This asymmetry is also apparent in the relationship between Headache and Pain in Figure 4.

**Figure 5.**
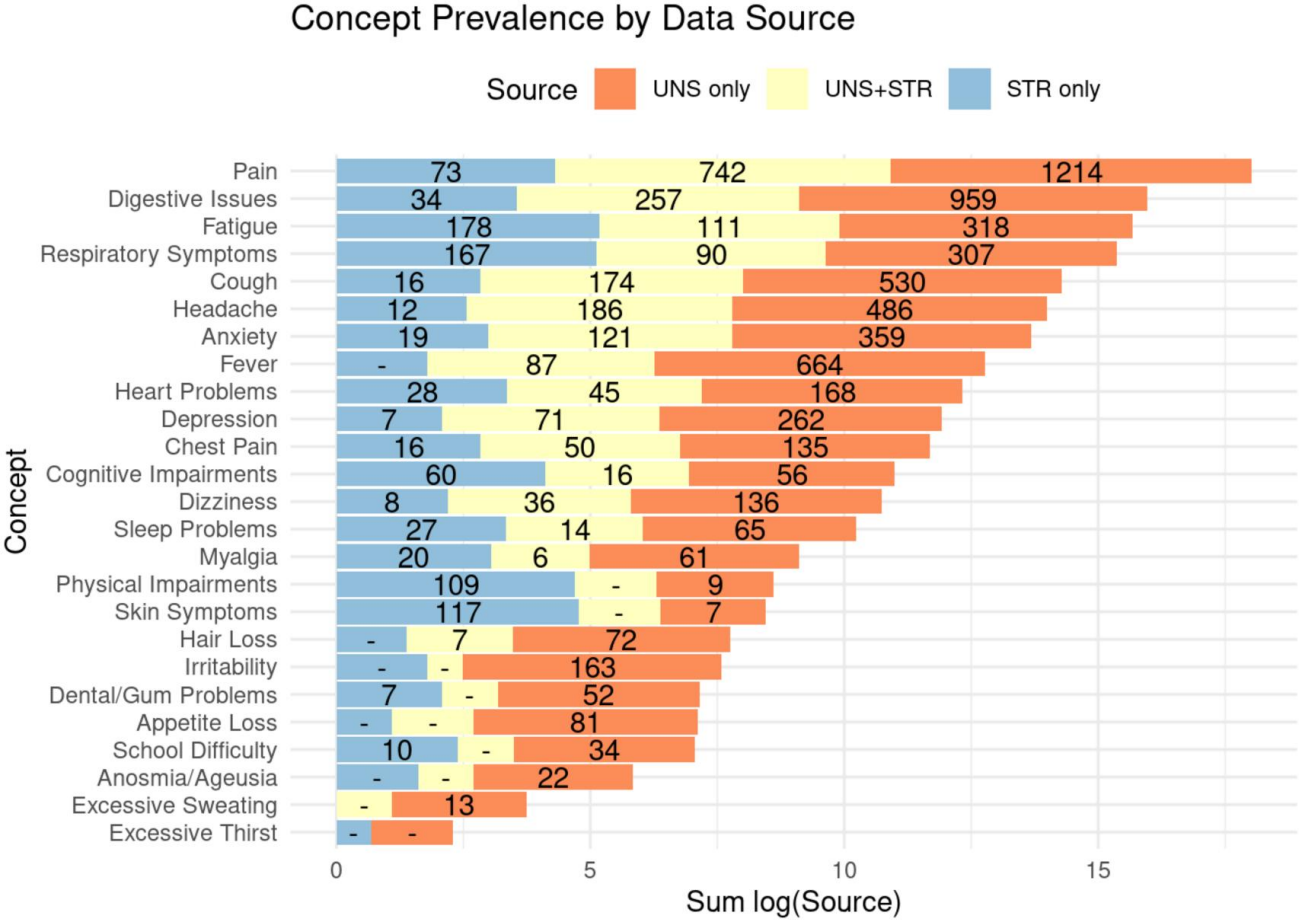
Patient counts for each concept from unstructured data only (UNS only), structured data only (STR only), or in both data sources (STR+UNS). Counts replaced by—have 5 or fewer patients.

A particularly revealing comparison of data from both sources is illustrated in Figure 5, which shows how patients are distributed within each concept based on the method used to assign the features. These data are ordered from the clinical concept found in the largest number of patients (pain) to that found in the fewest (Excessive Thirst). Patients identified using NLP alone are shown as the salmon segment of each bar, those identified only through structured EHR queries in the blue segment, and those identified from both sources in the yellow segment. Numbers within each segment indicate the number of patients (when > 5). Note that the horizontal scale is logarithmic to allow large differences to be represented within the chart. Crucially, for many concepts, more patients are identified through one or the other of the 2 sources and not common to both.

### Inter-site agreement

**Figure 6.**
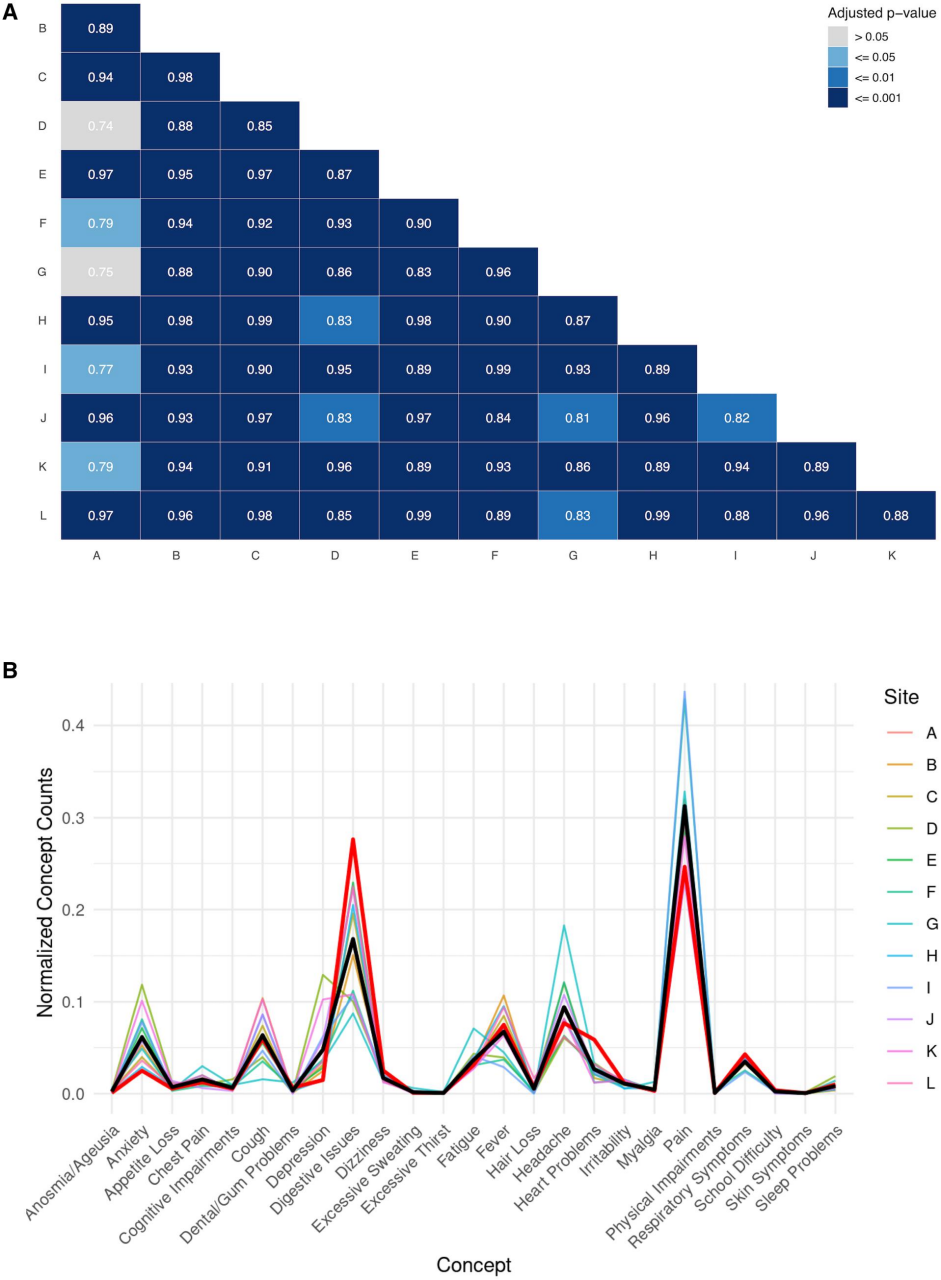
Frequency of concept mentions asserted present within each site (relative to total mentions per site). (A) Pair-wise correlations between sites with each cell showing the correlation in the number of mentions for each of the 25 concepts between the intersecting sites. The correlation is printed in each cell and cell shading reflects the probability of the correlation arising by change based on Bonferroni correction for 66 multiple comparisons. (B) Relative concept frequency by concept and site. Heavy black line is the average of all sites, heavy red line is Site A, the site that appears to be less related to other sites in terms of concept frequencies.

We examined inter-site agreement in terms of the relative frequency of *Present* term mentions in each of the 25 clinical concepts. Figure 6A presents pair-wise correlations in the relative frequency of term counts for each clinical concept. Overall, relative term frequency is strongly related. All but 2 of the 66 pairwise correlations are significant using Bonferroni corrections for multiple comparisons. Both non-significant correlations are associated with site “A,” which also participated in several other weaker inter-site correlations. Figure 6B shows relative term frequencies for each of the 25 concepts plotted separately for each site and illustrates some of the differences responsible for the lower correlations between site A and others. The heavy black line in the figure shows the average over all sites for each concept while lighter lines show individual sites. Site A is shown as a heavy red line in the figure. The relative term frequency for site A is an extremum for several of the concepts, for example, lowest for anxiety; highest for digestive issues; lowest for pain. Site A was one of only 2 sites where digestive issues were more frequently mentioned than pain, the overall most frequently mentioned concept. (See Supplemental Materials for a table of statistics from confusion matrices derived from each site. The per-site statistics are quite variable due to large differences in the number of SME responses to entities at each site [from 4 to 254].)

## Discussion

We developed a set of 25 symptoms and functional impairments that are associated with Long COVID.^7^^,^^10^^,^^15^ Each feature was in turn associated with a set of target terms that were identifiable through NER or RE search of unstructured notes data. Using an NLP pipeline comprising openly available components, licensed models,^13^ plus custom RE and word2vec models, we demonstrated the ability to identify feature-relevant terms and to determine with accuracy when terms were asserted to be present or not based on note context. (The RE term filters and assertion models developed here should generalize to other studies of COVID and Long COVID involving physician notes. Software to support SME screening of pipeline output may also be helpful as used here to assist in bootstrapping assertion labels from large untagged datasets and should generalize to areas other than COVID.)

After tuning the pipeline, we applied the pipeline to a sample of 5309 patients with presumptive Long COVID diagnosis and a matched sample of 5309 patients who experienced SARS-Cov-2 infection without evidence of Long COVID, as determined by a rules-based computable phenotype.^10^ Similarities and differences in patient cohorts and in the prevalence of features as identified from notes versus structured data were explored in several analyses. Additionally, we reported largely similar patterns in the relative frequency of Long COVID symptoms across the 12 participating sites despite difference in EHR systems and local practices. This similarity speaks to the generality of the approach despite moderate variation in note structure between sites.

Long COVID features assigned by our NLP pipeline were more prevalent among Long COVID patients. This lends support to the selection of terms and features for the NLP search. Many of the search terms are very commonly used in notes to review the history and status of a present illness. That there were substantial differences in the number of Long COVID as opposed to COVID patients to whom these symptoms were attributed and after adjusting for the number of notes suggests the composite assertion model was performing well at rejecting term mentions that were negated.

One of the most interesting results from the present study, and a major strength of the approach, is illustrated in Figure 5, which shows a substantial degree of separation in the patients identified through discrete EHR code search and NLP feature extraction. In many cases, the number of patients assigned a Long COVID feature by the NLP pipeline alone exceeded the number of patients assigned a feature from structured data alone or jointly from both analyses. One likely reason is that information in clinical notes documents patient clinical status regardless of whether it is related to a billable activity or observation, as is the case for many discrete elements. In addition, some Long COVID features may be poorly characterized by discrete EHR elements because appropriate coding is unavailable or not widely applied. In either case, the disparity underlines the extent to which analysis of notes data can extend our understanding of diseases when compared to structured data alone and the potential benefit of including both sources of data when possible.

### Challenges and limitations

A primary limitation of the current study is that it examined comparative feature prevalence (as opposed to incidence) in Long COVID versus COVID patients. For instance, Cognitive Impairment and Physical Impairment were expected to be better represented in notes data but emerged as more prevalent in structured data. In the structured data, these features were differentially associated with ADHD and mobility challenges respectively. It is likely that these were not incident but rather long-standing issues, unrelated to a SARS-Cov-2 infection. This limitation should be addressed in future analyses that identify incident features in a much larger number of COVID-19 patients compared to a control set of patients who present no evidence of having been infected with the SARS-Cov-2 virus.

Although we did not attempt a second full-scale SME review of the final pipeline output, 2 authors reviewed 400 tokens randomly sampled from notes not used in pipeline development. Results from this small sample support the conclusion that the adapted and tuned pipeline retained significantly greater accuracy that was observed for the original pre-tuned pipeline. For example, the terms like “depression” or “depressed” are often used in notes to refer to things like depressed blood flow or other reduced functional properties and not related to mood. This was common enough that we introduced RE rules to detect common non-relevant contexts. One indication that this may not have been a large confounding factor for depression is the comparatively strong correlation between structured and unstructured data for the term, and its strong correlation to Anxiety.

In the present analysis, entities not identified explicitly as *Present* were assumed to be *Absent*, making *Absent* effectively the default assertion. Consistent with this, pipeline changes that improved accuracy generally did so by correctly assigning *Present* to entities otherwise assumed *Absent*. Our rules for doing this were accurate and generalized well in our sample but are fundamentally heuristic and could generalize poorly to other samples. Perhaps the most promising alternative to approaches like ours is the application of large language models (LLMs) to extract symptoms and functional information from EHR notes.^17^^,^^18^ Studies using LLMs with smaller corpora (eg, reports and notes that have been carefully annotated) report encouraging results. However, prompt selection, which can strongly impact accuracy, is itself a heuristic process, and note quality can influence the likelihood of hallucinations. Augmenting language models to use chain of thought reasoning or external data and tools may address some of the challenges to applying LLMs at scale.^19^ Two areas of needed research related to applying LLMs to feature extraction from clinical data are (a) how to combine structured and unstructured sources of data as input to the LLM and (b) how to capture and represent the time course of disease progression and resolution within patients.

Applications involving NLP and machine learning are subject to potential biases. Bias can be introduced in AI/ML studies by inadequately sampling diverse populations when designing a study. In the current study, we used propensity matching to balance the number of patients in our 2 primary cohorts for sex, race, and ethnicity. Beyond these basic measures to control our study samples, we did not introduce additional factors such as socioeconomic or geographic information largely because it would too severely limit the sample size.

The current study chose 25 specific concepts that are known from existing literature to be associated with Long COVID in both adults and children.^20^^,^^21^ There are of course many other symptoms and factors that were not included in the present analysis, but may be important features or modifiers. For example, COVID vaccinations reduce the likelihood of Long COVID in children and could be included as an additional feature derived from notes as well as structured data. However, the current patient sample would not be ideal for exploring the impact of vaccinations since it largely covers a period when vaccines were unavailable or being introduced to different ages of patients over time.

## Conclusion

The current pipeline performed well in identifying Long COVID-related features from pediatric clinical notes. Crucially, while many patients were assigned the same features via both NLP analysis of notes, and queries designed to locate those features in structured data, an even larger number were not identified through structured data. Similarly, though to a lesser extent, patients presenting with Long COVID concepts based on structured data were often not those assigned the same feature from notes. These data clearly support the importance of incorporating NLP methodology into the design of computable phenotypes to more readily and accurately identify patients with Long COVID.

Perhaps the most significant limitation of this work is that it considered only the prevalence of Long COVID-related features during a critical period following the patients’ initial SARS-Cov-2 infection. This limitation should be addressed in future analyses that identify incident features in a much larger number of COVID-19 patients compared to a control set of patients who present no evidence of having been infected with the SARS-Cov-2 virus.

## Supplementary Material

ooaf089_Supplementary_Data

## Data Availability

The results reported here are based on detailed individual-level patient data compiled as part of the RECOVER Program. Due to the high risk of reidentification based on the number of unique patterns in the data, particularly physician notes data, patient privacy regulations prohibit us from releasing the data publicly. The data are maintained in a secure enclave, with access managed by the program coordinating center to remain compliant with regulatory and program requirements. Please direct requests to access the data, either for reproduction of the work reported here or for other purposes, to recover@chop.edu.
