# Supplementary material for: A natural language processing pipeline for identifying pediatric long COVID symptoms and functional impacts in freeform clinical notes: a RECOVER study
Source: JAMIA Open. 2025 Sep 4;8(5):ooaf089. doi: 10.1093/jamiaopen/ooaf089 (PMC12409404; doi:10.1093/jamiaopen/ooaf089)
Supplement: ooaf089_Supplementary_Data [file ooaf089_supplementary_data.docx]

# Supplemental Material

## Temporal Distribution of Patients and Notes

Our patient sample was drawn from the period between March 2020 and August 2022. Notes collected from these patients were drawn from the period January 2020, to September 2022. Note that imputed index dates (date of earliest COVID diagnosis for Long COVID patients who did not have a prior COVID diagnosis) could extend back to January 2020.

Figure S1. Distribution of patients within the data collection period arranged by the date of earliest COVID diagnosis (*index_date*).

Figure S2. Distribution of notes over the data collection period by note date.

## NLP associated terms for Long COVID features

| Feature | Associated Terms |
| --- | --- |
| AnosmiaAgeusia | Anosmia, paraosmia, dysgeusia, olfactory dysfunction, sense of smell altered, absence of/loss of/diminished sense of smell/taste, change in smell/taste, smell/taste alteration |
| Anxiety | Adjustment disorder, anxiety/anxious attack/disorder/state/mood, excessive fear, feeling fearful/scared/nervous, panic attack, trouble/difficulty with relaxing |
| AppetiteLoss | Anorexia [not nervosa], early satiety, little/no/limited/poor appetite, losing/lacking appetite, doesn't want to eat, little/no/lack of interesting in food/eating |
| ChestPain | Chest pain, pain in chest |
| Cough | Barking/chronic/persistent/mild/moderate/sever/frequent/hacking cough, coughing during/while/when, frequently coughing, coughing frequently |
| DentalGumProblems | Problems/issues in/with teeth/gums, teeth/tooth/gum problems/issues, poor/bad gums, cracks/chips in teeth, mouth sores, dry mouth, jaw/tooth pain, pain in jaw/tooth/teeth, swollen/bleeding gums |
| Depression | Unhappy, depressed/depression [not screening, not cardiac], suicidal, feeling sad/worthless/helpless/like a failure, no reason to live, nothing to look forward to |
| DigestiveIssues | Nausea, vomiting, upset stomach, poor appetite, throwing up, retching, emesis, sick to stomach, queasy, regurgitation [not cardiac], loose stool, diarrhea, difficulty/problems defecating, constipation, reflux [not urine] |
| Dizziness | Dizzy, vertigo, room is/was spinning |
| ExcessiveSweating | Excessive/profuse sweating, sweating too much/excessively/profusely, night sweats, hyperhidrosis, diaphoresis |
| Fatigue | Feels exhausted/tired/weak/worn out, fatigued, lethargic, listless, malaise, run-down, general/overall/pervasive/intermittent weakness, weary, sluggish, exhaustion, easily/mentally/physically/totally/too tired/drained, not enough/out of energy, tires quickly/with exercise/all the time |
| Fever | [not hay] fever/feverish, pyrexia, raised/elevated/high body temperature, shivering, temperature spike/raised/elevated/high, febrile, feeling hot, rigors |
| HairLoss | Loss/shedding of hair, hair loss/shedding, losing hair, alopecia, telogen effluvium |
| Headache | Headache, head aches, migraine [not team/program], HA, migrainous |
| HeartProblems | Palpitations, tachycardia, heart beating fast/hard, heart racing/pounding, syncope, long qt |
| Irritability | holding breath, irritability, irritable [not bowel], fussy, aggressivity |
| Myalgia | Myalgia, muscle ache/pain, muscles hurt, pain/ache in muscle |
| Pain | Pain [not clinic/center], stomachache, headache, aching/cramping/sore/tender |
| RespiratorySymptoms | Dyspnea, difficult/shallow/labored/rapid breathing, sob, shortness/out of breath, unable/struggling to breathe, wheezing, panting, gasping for/runs out of air, winded, tight chest, chest feels tight, restricted airflow, asthma, rhonchi, work of breathing |
| SkinSymptoms | Rash, [not mouth/throat] pruritis, itching skin, skin itches, erythema, swelling |
| CognitiveImpairments | Brain fog, attention/concentration/memory/focus issues/problems, difficulty/trouble/harder time/issues with concentrating/remembering/attention/focus, decreased/lower attention/concentration/remembering/focus |
| PhysicalImpairments | Trouble/difficulty/problems/hard time climbing stairs/walking/running/exercise playing/dressing, functional limitations, postural control |
| SchoolDifficulty | Missing/dropping/failing/difficulties at/with/in school/class, below average/low/failing/falling/worsening grades, grades are dropping/falling/worsening, academic/school/educational issues/problems/difficulties, not doing well/doing poorly at school |
| SleepProblems | Insomnia, not sleeping very well/enough/much/at all, trouble/difficulties/hard time sleeping, parasomnia, nightmares, night terrors |

## Code Sets

### Excluded Providers

| Concept ID | Concept Name |
| --- | --- |
| 0 | No matching concept |
| 903231 | Behavior Technician |
| 903254 | Orthopedic Assistant |
| 38003633 | Behavioral Health & Social Service Providers, Behav. Analyst |
| 38003656 | Behavioral Health & Social Service Providers, Social Worker |
| 38003657 | Clinical Social Worker |
| 38003684 | Dental Hygienist |
| 38003688 | Nutritionist |
| 38003690 | Registered Dietitian |
| 38003691 | Registered Pediatric Dietitian |
| 38003692 | Registered Renal Dietitian |
| 38003694 | Registered Dietetic Technician |
| 38003709 | Ophthalmic Technician |
| 38003716 | Registered Nurse |
| 38003722 | Registered Community Health Nurse |
| 38003733 | Registered Gerontology Nurse |
| 38003743 | Registered Neonatal Intensive Care Nurse |
| 38003749 | Registered Pediatric Nurse |
| 38003752 | Registered Psych / Mental Health Nurse |
| 38003763 | Registered Women's Health Care Ambulatory Nurse |
| 38003764 | Registered Obstetric High-Risk Nurse |
| 38003766 | Registered Occupational Health Nurse |
| 38003772 | Licensed Practical Nurse |
| 38003776 | Genetic Counselor |
| 38003780 | Acupuncturist |
| 38003782 | Interpreter |
| 38003802 | Lactation Consultant |
| 38003810 | Pharmacist |
| 38003815 | Pharmacist Clinician (PhC) / Clinical Pharmacy Specialist |
| 38003816 | Pharmacotherapy Pharmacist |
| 38003819 | Pharmacy Technician |
| 38004038 | Art Therapist |
| 38004065 | Athletic Trainer |
| 38004068 | Massage Therapist |
| 38004069 | Recreation Therapist |
| 38004070 | Music Therapist |
| 38004087 | Pediatrics Occupational Therapist |
| 38004090 | Certified Respiratory Therapist |
| 38004104 | Registered Respiratory Therapist |
| 38004119 | Audiologist |
| 38004122 | Speech Language and Hearing Specialist / Technologist |
| 38004123 | Audiology Assistant |
| 38004125 | Speech, Language & Hearing Providers, SLP |
| 38004146 | Cardiology Technician |
| 38004155 | Technologist, Technician, Other Technical Service Provider |
| 38004188 | EEG Technician |
| 38004228 | Ambulatory Infusion Therapy Clinic / Center |
| 38004250 | Ambulatory Radiology Clinic / Center |
| 38004294 | Clinical Medical Laboratory |
| 38004439 | Nurse's Aide |
| 38004489 | Audiology |
| 38004490 | Physical Therapist |
| 38004492 | Occupational Therapy |
| 38004499 | Social Worker |
| 38004515 | Hospital |
| 38004520 | Pharmacy |
| 38004689 | Public Health or Welfare Agency |
| 38004694 | Registered Dietitian or Nutrition Professional |
| 43125857 | Dental Therapist |
| 44814649 | Other |

## EHR Code Sets for Long COVID features

Github repository for supplemental data and code.

<https://github.com/RECOVER-Coordinating-Center/pediatric_nlp_manuscript_1>

## John Snow Labs Pipeline Components

1. Sentence detection and splitting (*DocumentAssembler*, *SentenceDetector* with split length set to 150)
2. Sentence tokenization (*Tokenizer*)
3. Word embeddings (*embeddings_clinical* version 2.4.0)
4. Three healthcare-specific NER models: *ner_jsl* (version 4.2.0), *jsl_ner_wip_clinical* (version 3.0.0), and *bert_token_classifier_ner_jsl* (version 3.4.0). The outputs of the three NER models were merged into a single NER stream for processing by the assertion model. In cases where the three NER models disagreed on how to classify an entity, the output of the model that had the highest confidence level was used. In case of a tie, the output of the earlier model in the list above was used.
5. The *assertion_jsl_augmented* model (version 4.1.0) was then used to process the combined NER output, adding assertion status (*Present*, *Absent*, *Hypothetical*, etc.).

## Assertion Model and Pipeline Tuning


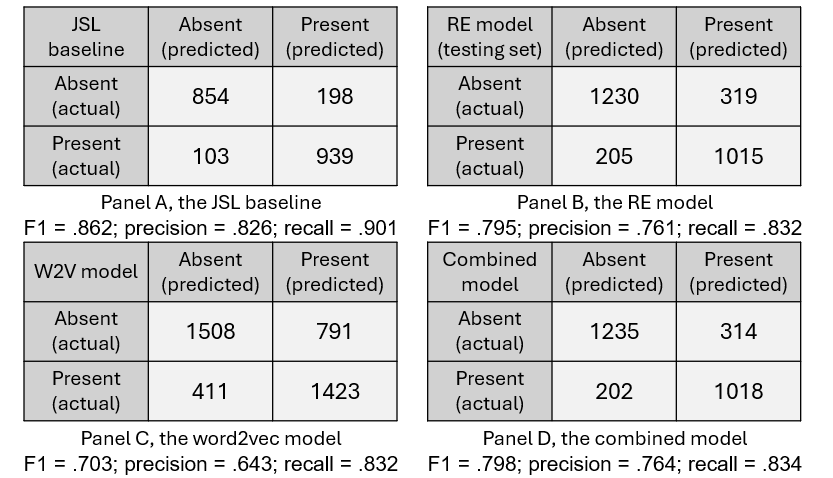


**Figure S3**. Performance of four models for predicting entity assertions.

We developed and tuned a composite assertion measure that used RE and wave2vec *Present/Absent* assertion models in combination with all JSL assertions when available. The RE model searched before and after each recognized entity for terms indicating either negation or confirmation that the entity was *Present*. Using a training set of 1364 SME responses, we iteratively adjusted the pre- and post-entity RE search range and expanded the list of negation/confirmation terms to find an optimal range. While doing this, we also modified the list of expressions the RE was using, e.g., to differentiate low-grade fevers from low grades in school.

To tune the word2vec assertion model we used 10-fold cross validation for exploring the range of tokens pre- and post-entity whose word vectors were averaged to form basis vectors for an assertion decision. The model used three basis vectors assigned *Present*, *Absent*, or *Hypothetical* by SMEs that were formed by averaging word vectors in the training fold for tokens within the range being tested. When compared to a probe vector in the test fold computed as the average of token vectors surrounding a target entity, the basis vector with the highest cosine similarity to the probe vector would determine the word2vec assertion: *Present* if the *Present* basis vector was most similar; *Absent* otherwise. The amount of weight given the *Hypothetical* assertion vector was also reduced after testing.

Figure S4 shows confusion matrices and performance metrics (accuracy, precision, recall, and F1) for four NLP assertion models, while figure S4 illustrates the partitions of the SME data set, beginning with the initial full set of response (n=4586), then the filtered final set of relevant responses (n=4133), from which the training (n=1364) and testing (n=2769) sets were drawn. Of the final SME set, 2094 were marked as either present or absent by both the JSL model and the SMEs. In figure S3, panel a) shows the performance of the baseline JSL assertion model was very good when only considering entities in the present/absent only set (N = 2094; F1 = .862; precision = .826; recall = .901). However, many entities classified by the JSL model as neither present nor absent or not classified at all were classified by SMEs as either present or absent. Panel b) shows our best performing RE assertion measure, developed using entities in the final SME set (N=4113). The RE model used 60 characters of prior context and 7 characters of subsequent context for its main search for negation terms, as this amount of context produced the best results on the training set (testing set N=2769 F1 = .795; precision = .761; recall = .832). It uses fifteen checks for negation terms and, if a negation term is found, three checks for present assertion terms. For the same entities, panel c) shows the performance of a phrase embedding model based on word embeddings from our word2vec models (N=4133 F1 = .703; precision = .643; recall = .832). These embeddings were developed by averaging the embeddings for all tokens within 70 characters before and after the target term in a sample of the SME assertions that were classified as either ‘present’, ‘absent’, or ‘hypothetical’ and testing using 10-fold cross-validation. The predictor looks at whether the 70 characters before and after a target term are closer to the ‘present’ embedding, ‘absent’ embedding, or ‘hypothetical’ embedding; .25 is subtracted from the similarity distance with the ‘hypothetical’ embedding, and if it still the most similar, an ‘absent’ assertion is assigned.


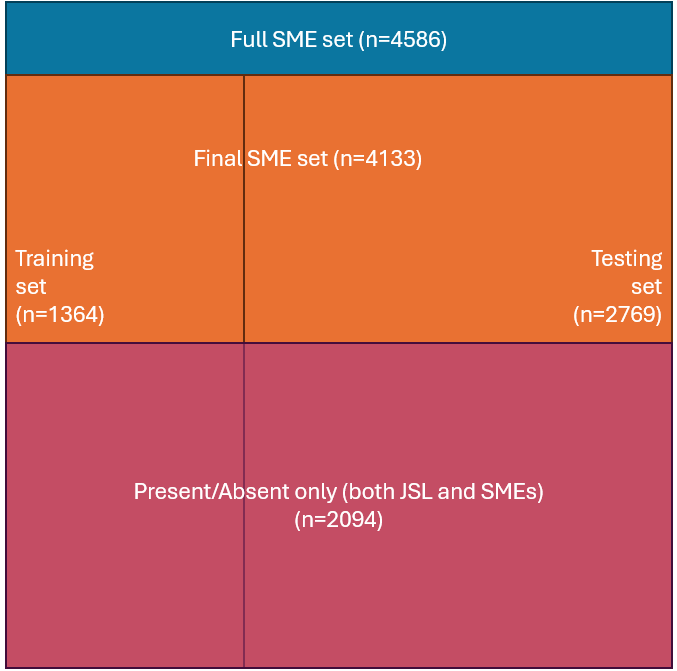


**Figure S4** – Partitioning of the full dataset illustrating the size relationships among test, training, and evaluation sets.

Our best assertion performance (testing set N=2769 F1 = .798; precision = .764; recall = .834), shown in panel d) was obtained with a composite model that used a majority call when all three models provided an assertion. In cases where the JSL model did not assign an assertion, we used the output of the RE model.

This composite assertion model was used in analysis of the full set of 48,287 notes from 10618 patients. Ninety-six of the Long COVID patients in the full dataset also contributed notes to our model evaluation dataset. Consequently, 574 terms reviewed by the SMEs were also used in the final analysis.

## ScreenTool

To facilitate the SME review, a user interface was developed that presented each token in the context of a 200-character note extract. As shown in Figure S5, the interface permitted SMEs to indicate the relevance (binary choice) of the highlighted term to a Long COVID feature (termed “Concept” in the interface) and assign one of five assertion tags to the feature. If there is insufficient information available in the note context to assign an assertion tag, SMEs are permitted to abstain from providing one, in which case an assertion label of “noCall” is assigned to indicate that no specific assertion was obtained on the trial. The noCall label is also used for cases where the NER models did not identify an entity for the JSL assertion model. Terms classified as “Not Relevant” by SMEs are assigned an assertion tag of “badCall” for analysis purposes.


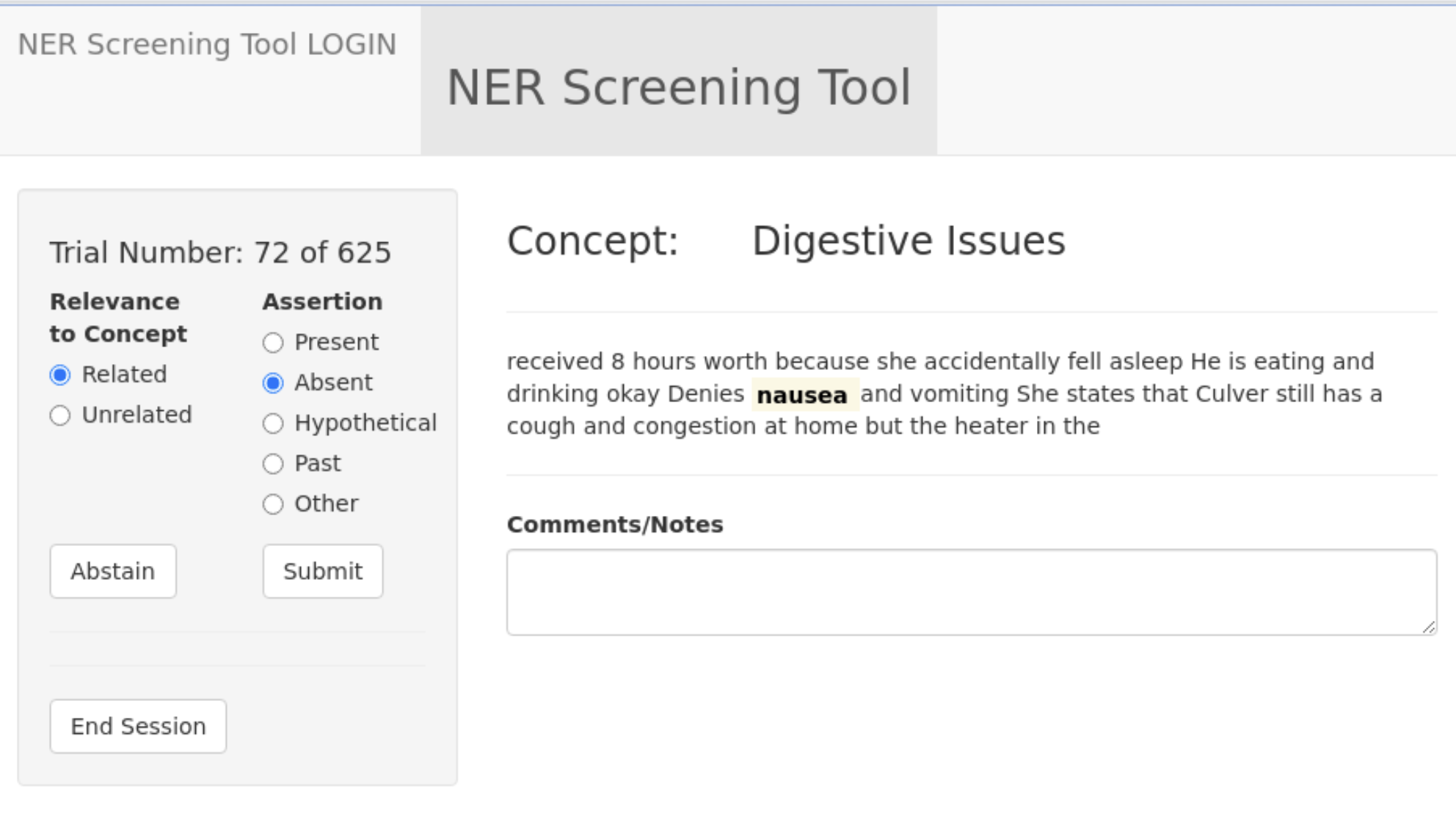


Figure S5. User interface for SMEs to score the NER and Assertion status of pipeline output.

Figure 4. Evaluation of three independent assertion models (a - c) and one composite model (d) which used a majority vote of the three independent models with fallback logic when the JSL assertion model did not assign a label.

## Pipeline accuracy across sites

The following estimates of accuracy by site are based on the pooled responses of all SMEs from the initial pipeline evaluation as well as responses from the two authors who reviewed 400 items from the final pipeline output. As the table below illustrates, there is substantial variability in the number of observations varies widely from as few as 4 (sites A and J) to as high as 264 for site C. This is the number of entities from each site for which we recorded an SME assertion to use as the ground truth comparison to the assertion generated by the pipeline.

## Table of CM statistics by site

| **Site Statistics Using all SME Responses** | | | | | | | |
| --- | --- | --- | --- | --- | --- | --- | --- |
| **Site** | **N** | **% Cor.** | **FPR** | **Precision** | **TPR Recall** | **F1** | ***p*** |
| A | 4 | 50.00 | 0.000 | 1.000 | 0.333 | 0.500 | 0.262 |
| B | 119 | 80.67 | 0.283 | 0.712 | 0.875 | 0.785 | <0.001 |
| C | 264 | 76.14 | 0.391 | 0.698 | 0.852 | 0.768 | <0.001 |
| D | 38 | 84.21 | 0.231 | 0.880 | 0.880 | 0.880 | 0.016 |
| E | 27 | 88.89 | 0.133 | 0.833 | 0.909 | 0.870 | <0.001 |
| F | 248 | 75.00 | 0.368 | 0.725 | 0.817 | 0.769 | <0.001 |
| G | 37 | 72.97 | 0.350 | 0.588 | 0.769 | 0.667 | <0.001 |
| H | 46 | 78.26 | 0.389 | 0.750 | 0.875 | 0.808 | <0.001 |
| I | 13 | 69.23 | 0.600 | 0.625 | 0.833 | 0.714 | 0.104 |
| J | 4 | 100.00 | 0.000 | 1.000 | 1.000 | 1.000 | 0.578 |
| K | 125 | 81.60 | 0.190 | 0.836 | 0.824 | 0.830 | <0.001 |
| L | 26 | 76.92 | 0.333 | 0.545 | 0.857 | 0.667 | <0.001 |
